# Preliminary Multi-Omics Insights into Green Alternatives to Antibiotics: Effects of *Pulsatilla chinensis*, *Acer truncatum*, and *Clostridium butyricum* on Gut Health and Metabolic Regulation in Chickens

**DOI:** 10.3390/ani15091262

**Published:** 2025-04-29

**Authors:** Lin Sun, Zhijun Wang, Shidi Qin, Chunhong Liang, Ayong Zhao, Ke He

**Affiliations:** 1 Key Laboratory of Applied Technology on Green-Eco-Healthy Animal Husbandry of Zhejiang Province, Hangzhou 311300, China; sunnylinnnn@163.com (L.S.); zhijunwang@zafu.edu.cn (Z.W.); qsd1261362977@163.com (S.Q.); m17342015356@163.com (C.L.); zay503@zafu.edu.cn (A.Z.); 2 Zhejiang Provincial Engineering Laboratory for Animal Health Inspection & Internet Technology, College of Animal Science and Technology & College of Veterinary Medicine, Zhejiang Agriculture and Forestry University, Hangzhou 311300, China

**Keywords:** chicken, antibiotic alternatives, gut microbiota, traditional Chinese medicine

## Abstract

Antibiotic resistance has become a global health concern, driving the need for sustainable alternatives in animal husbandry. This study explores the potential of natural feed additives as a viable solution to enhance poultry growth and health while reducing reliance on antibiotics. Chinese herbal medicines and probiotics have been widely studied as green, healthy, and safe antibiotic alternatives in livestock and poultry production. A total of 120 chickens were randomly divided into four groups: a control group and three treatment groups supplemented with 1% *Pulsatilla chinensis* powder, 3% fresh *Acer truncatum*, or 1% *Clostridium butyricum*. The results showed that *Pulsatilla chinensis* powder significantly increased gamma-glutamylcysteine (*p* < 0.05), UDP-N-acetylglucosamine (*p* < 0.05), tyramine (*p* < 0.01), and leucine (*p* < 0.05). *Acer truncatum* notably altered cecal metabolites, including L-tyrosine (*p* < 0.05), α-ketoisovaleric acid (*p* < 0.01), myristoleic acid (*p* < 0.01), glutathione (*p* < 0.05), and PGA1 (*p* < 0.05). *Clostridium butyricum* modified cecal metabolites such as L-glutamine (*p* < 0.05), riboflavin (*p* < 0.05), L-Carnitine (*p* < 0.05), ergocalciferol (*p* < 0.01), and α-tocotrienol (*p* < 0.05).

## Simple Summary

This study examines how Pulsatilla chinensis, Acer truncatum, and Clostridium butyricum work as natural feed additives for Jianmen-guan chickens. Chickens were randomly divided into four groups: control group, 1% Pulsatilla powder, 3% fresh Acer truncatum, and 1% Clostridium butyricum. Pulsatilla chinensis boosted antioxidant capacity by upregulating glutathione metabolism genes. Acer truncatum changed tyrosine and lipid metabolism, while Clostridium butyricum enhanced immunity and altered gut microbiota. All additives improved gut bacteria and growth performance, suggesting their potential for improving poultry health.

## 1. Introduction

The overuse of antibiotics in poultry production has led to the emergence of antibiotic-resistant bacteria, posing a significant threat to both animal and human health [1]. To address this issue, natural additives such as herbal medicines and probiotics have been explored as green alternatives to antibiotics. A study has found that traditional Chinese medicine (TCM) demonstrates unique advantages in treating drug-resistant bacterial infections by modulating the gut microbial microenvironment, offering new strategies to unravel the interactions between microbiota and pathogen resistance [2]. Among these, *Pulsatilla chinensis*, *Acer truncatum*, and *Clostridium butyricum* have shown unique potential due to their specific bioactive properties. This study aims to evaluate their combined effects as a sustainable alternative to antibiotics in poultry production.

*Pulsatilla chinensis* and *Acer truncatum* were used as herbal feed additives in this study. *Pulsatilla chinensis* is known for its antimicrobial, anti-inflammatory, and immunomodulatory properties, with potential as an antibiotic alternative. *Acer truncatum*, a local economic crop in the Jianmen-guan region, is rich in polyphenolic compounds with antioxidant and anti-inflammatory activities. Both TCMs are green and safe, offering complementary benefits such as immune regulation and oxidative damage protection. *Pulsatilla chinensis* is a TCM with heat-clearing, detoxifying, and antidiarrheal properties, widely used in treating schistosomiasis and inflammatory bacterial infections [3]. Research has shown that adding 1.5% of a Chinese herbal medicine compound preparation made from *Pulsatilla chinensis* to the diet promotes growth and enhances the production performance of broiler chickens. During the 42-day experimental period, the average daily gain increased by 7.38%, and the feed-to-gain ratio (F/G) decreased by 6.25 [4]. Adding 0.5% of *Pulsatilla chinensis* powder to the diet of laying hens in the later stages of egg production can improve their glucose and lipid metabolism, enhance their immune and antioxidant functions, and strengthen the mucosal barrier function of the small intestine [5]. In sheep farming, the administration of 10 mL of the preparation per lamb, twice a day, can be used to treat colibacillosis in sheep [6]. *Acer truncatum*, native to China, is commonly used to treat skin itching and cracking, and has potential inhibitory effects on various skin inflammations [7,8]. In animal husbandry, research has shown that, compared with the control group, supplementing the diet with 5% air-dried *Acer truncatum* leaves significantly improved the ADG and average daily feed intake (ADFI) of piglets (*p* < 0.05), reduced the diarrhea rate (*p* < 0.05), improved their serum biochemical indicators, and enhanced their immune capacity [9]. Additionally, 0.3% ATL extract can regulate intestinal microbiota, enhance antioxidant capacity, and alleviate lipopolysaccharide-induced inflammation in broilers [10].

*Clostridium butyricum* is a strictly anaerobic spore-forming bacillus, commonly found as a commensal in human and animal intestines, with non-toxic strains used as probiotics [11]. A study found that supplementing the basal diet with 1 × 10^9^ colony-forming units (CFU)/kg of *Clostridium butyricum* resulted in significantly higher serum total antioxidant capacity (t-AOC), superoxide dismutase (SOD) activity, and glutathione peroxidase (GSH-Px) activity at day 42 compared to the control group (*p* < 0.05) [12]. Supplementation with 1.0 × 10⁹ CFU/tonne (t) of *Clostridium butyricum* was found to maintain intestinal barrier function, reduce inflammatory responses, and increase short-chain fatty acid concentrations in the ileum and cecum [13]. *Clostridium butyricum* was able to decrease cytokine levels (IFN-γ, IL-1β, IL-8, and TNF-α) in intestinal tissues and epithelial cells through TLR4-, MyD88-, and NF-κB-dependent pathways, alter the intestinal microbial composition, and inhibit Salmonella [14]. In piglet feeding experiments conducted over a 0–21-day period, *Clostridium butyricum* altered intestinal metabolites, mainly involving changes in the metabolic pathways of citrulline, dicarboxylic acids, branched-chain amino acids, and tryptophan, which can provide potential benefits for intestinal health [15].

The abuse of antibiotics has led to the spread of superbugs, severely impacting human health [16]. JIC is a regional brand from Guangyuan City, Sichuan Province, China. Characterized by its native chicken lineage, delicious flavor, and eco-friendly attributes, it is highly favored by consumers but is not exempt from this problem. We hypothesized that *Pulsatilla chinensis*, *Acer truncatum*, and *Clostridium butyricum* could serve as effective green alternatives to antibiotics by improving growth performance, gut health, and metabolic function in poultry. This study aims to evaluate their combined effects as sustainable alternatives to antibiotics in poultry production, contributing to the development of environmentally friendly, healthier, and more efficient local chicken farming practices that meet consumer demands for high-quality and safe animal products.

## 2. Materials and Methods

### 2.1. Animal Model Treatment and Sample Collection

A total of 120 healthy one-day-old male chicks with similar body weights (approximately 40 g) were randomly assigned to 4 treatment groups (with 30 chickens in each group, 3 replicates per group, and 10 chickens per replicate) to ensure consistency and comparability. All chicks were reared in cage systems under standard management conditions: the temperature was maintained at a constant 32 °C for the first week, gradually decreasing by 2 °C per week until it reached ambient room temperature for the remainder of the rearing period, and the humidity in the chicken coop was controlled to remain between 50% and 70%. The experiment lasted for 150 days to evaluate the effects of different treatments on growth performance and health indicators. The experimental groups were designed as follows: (1) an untreated group (UT), fed a basal diet; (2) a Pulsatilla-treated group (PT), fed a basal diet supplemented with 1% Pulsatilla powder; (3) an *Acer truncatum*-treated group (AT), fed a basal diet supplemented with 3% fresh *Acer truncatum*; and a (4) *Clostridium butyricum*-treated group (CB), fed a basal diet supplemented with 1% *Clostridium butyricum* (commercial probiotic strain). The basal diet was formulated to meet the nutritional requirements of Jianmen-guan indigenous chicken (JIC). The composition and nutritional content of the basal diet are presented in Table 1.

Chickens in each group were housed separately in 4 m × 4 m pens and allowed to engage in free-range activity. Each chicken was provided with 0.25 kg of feed daily and allowed to eat ad libitum. The euthanasia method and tissue collection scheme were approved by the Animal Health Committee of Zhejiang Agricultural and Forestry University (Hangzhou, China) with approval number ZAFUAC202441.

### 2.2. Biometric Measurements and Sample Collection

At 150 days of age, chickens from each group (UT: *n* = 6; PT: *n* = 6; AT: *n* = 7; CB: *n* = 7) were euthanized. The cecal contents were collected from the blind end of the cecum and immediately frozen in liquid nitrogen for 16S rRNA sequencing. The middle segment of the cecum was washed with sterile saline and snap-frozen for transcriptomic and metabolomic analyses. Biometric measurements (e.g., body weight, muscle mass) were recorded as Supplementary Data.

### 2.3. Transcriptome Analysis

Total RNA was extracted from cecal tissue using the TRIzol Reagent (Thermo Fisher Scientific, Carlsbad, CA, USA), following the manufacturer’s instructions. RNA quality was assessed using the Agilent Bioanalyzer 2100 system (Agilent Technologies, Santa Clara, CA, USA), and only samples with an RNA Integrity Number (RIN) ≥ 8.0 were used for subsequent library preparation. PolyA mRNA was enriched from total RNA using oligo(dT) beads, and the mRNA was fragmented to approximately 300 bp. cDNA synthesis and library construction were performed using the NEBNext Ultra II RNA Library Prep Kit for Illumina (New England Biolabs, Ipswich, MA, USA), following the manufacturer’s protocol. The quality of the libraries was assessed using the Agilent Bioanalyzer 2100 system, and quantification was performed using the Qubit Fluorometer (Thermo Fisher Scientific, Carlsbad, CA, USA). Libraries were then subjected to paired-end sequencing (2 × 150 bp) on the Illumina NovaSeq 6000 platform at Personal Biotechnology Co., Ltd. (Shanghai, China). The reference genome used was Gallus_gallus.GRCg6a.dna.toplevel.fa, which was obtained from the Ensembl database at http://ftp.ensembl.org/pub/release-105/fasta/gallus_gallus/dna accessed on 12 October 2021.

To ensure the comparability of gene expression levels across different genes and samples, the expression levels were normalized using fragments per kilobase of transcript per million mapped reads (FPKM) [16]. Principal Component Analysis (PCA) was performed to analyze the expression amount, and DESeq was used to examine differences in gene expression and obtain data regarding fold change and the significance *p*-value for each gene. The differences between the control and treatment groups were then filtered based on the criteria: fold change > 1.5 and *p*-value < 0.05. Then, GO (Gene Ontology, http://geneontology.org/ accessed on 12 October 2021) and KEGG (Kyoto Encyclopedia of Genes and Genomes, https://www.kegg.jp/ accessed on 12 October 2021) enrichment analysis were applied to establish gene function.

### 2.4. LC-MS Non-Targeted Metabolic Analysis

Cecal tissue was used as a sample. We applied metabolite extraction, quality control (QC) preparation, LC-MS/MS analysis, and mass spectrometry analysis to obtain metabolomic profiles. Next, the metabolites were identified by matching their retention times and molecular masses (with a mass error threshold of <10 ppm), and we compared MS/MS spectra with those in a locally built database and public databases (HumanMetabolome Database (http://www.hmdb.ca accessed on 12 October 2021), Metlin (http://metlin.scripps.edu accessed on 12 October 2021), massbank (http://www.massbank.jp/ accessed on 12 October 2021), mzclound (https://www.mzcloud.org accessed on 12 October 2021)). The identification level was set at Level 2 or higher.

Subsequently, abundance analysis of the identified metabolites was performed. Pairwise comparisons between the treatment and control groups, using multivariate statistical analysis, were also conducted. Potential differential metabolites were identified based on the criteria of critical Variable Importance in Projection (VIP) > 1.0 and fold change > 1.5. Furthermore, potential differentially expressed metabolites were identified, and KEGG enrichment analysis was performed on these metabolites to explore their biological functions.

### 2.5. 16 S rRNA Sequencing

Cecal content samples were used as templates for amplifying the bacterial 16S rRNA gene hypervariable V3-V4 region. Amplification was performed using the universal primer pair of F: ACTCCTACGGGAGGCAGCA and R: GGACTACHVGGGTWTCTAAT.

For the extraction of DNA from cecal content, the Universal Genomic DNA Kit was utilized, following the manufacturer’s instructions meticulously. The ratio of absorbance at 260 nm to 280 nm (A260/A280) was detected to verify the absence of protein contamination (ideally, a ratio between 1.8 and 2.0 indicates good purity) and check the absorbance at 260 nm to quantify the DNA yield. Additionally, the integrity of the DNA was visually inspected on an agarose gel to confirm the presence of intact high-molecular-weight DNA bands. Community DNA fragments were sequenced using the Illumina platform (Illumina-NovaSeq-6000) with a paired-end approach. The DADA2 pipeline [17] was employed to process the data, and this involved the following key steps: adapter trimming, quality filtering, denoising, and chimera removal. Notably, the DADA2 algorithm clusters sequences with 100% similarity, resulting in the generation of amplicon sequence variants (ASVs) that are free of errors and redundancy. We then compared data with the Greengenes database (Rlease 13.8, https://ftp.microbio.me/greengenes_release/current/ accessed on 12 October 2021) [18]. Alpha diversity was assessed using the Chao1 estimator, Simpson index, Shannon–Wiener diversity index, and Faith’s Phylogenetic Diversity. Beta diversity was analyzed using Bray–Curtis distance to determine inter-group differences. For linear discriminant analysis, an LDA cutoff of 2.0 was applied to identify significant taxa contributing to group differences. Venn diagrams were constructed using the VennDiagram package in R (version 4.3.1) to illustrate the number of core and unique species across groups. Taxonomic composition was visualized through a heatmap generated based on the top 20 most abundant genera. The linear discriminant analysis effect size (LEfSe) analysis was employed to identify the impact of categorical variables on inter-group differences. An LDA threshold of 2.0 was used to determine the effect size of the taxa. An abundance filtering threshold of 0.001 was applied to ensure that only taxa with sufficient representation were included in the analysis.

Analyses of 16S, transcriptomic, and metabolomic data were performed using the GenesCloud online platform (https://www.genescloud.cn/ accessed on 12 October 2021).

### 2.6. GO and KEGG Annotation and Enrichment

The detected genes and proteins were functionally annotated in the Gene Ontology (GO, http://www.geneontology.org accessed on 12 October 2021) and Kyoto Encyclopedia of Genes and Genomes (KEGG, http://www.genome.jp/kegg accessed on 12 October 2021) databases. Enrichment analysis was carried out using the software Goatools (version 1.3.0) and Python scipy (version 1.11.1), and the BH method was used to perform multiple tests in order to control the calculation of the false-positive rate, and the *p*-value < 0.05 was considered to be significantly enriched.

### 2.7. Association Analysis

First, an association analysis between the transcriptomic and metabolomic results was conducted. Information on differentially expressed metabolites was obtained from the KEGG Small Molecule Database (https://www.kegg.jp/dbget-bin/www_bfind?compound accessed on 12 October 2021) and detailed information on related transcripts, including ID, name, involved metabolic pathways, and related enzymes was obtained from the KEGG Orthology database (https://www.kegg.jp/kegg/ko.html accessed on 12 October 2021). Then, based on the results of the differential analysis, differentially expressed metabolites and their corresponding differentially expressed transcripts were screened. These differentially expressed metabolites and transcripts were paired, and their fold changes in expression levels were presented. We also explored the commonly enriched pathways based on the differentially expressed metabolites and the single-omics enrichment analysis results of differentially expressed genes (DEGs).

Secondly, correlation analysis was performed between cecal 16S rRNA and metabolome results. PCA and Co-Inertia Analysis (CIA) were conducted by applying the R package “ade4” to assess genus-level diversity composition and metabolites. A two-dimensional CIA ordination plot was generated. Spearman rank correlation coefficients were calculated between metabolome data and bacterial abundance. A heatmap was constructed based on the correlation coefficient matrix and validation results. An association network was built using correlations with |rho| > 0.8 and *p*-value < 0.01. The network was visualized using Cytoscape software (Version 3.9.1).

### 2.8. Statistical Analysis

Growth performance data were used to screen for body weight outliers prior to multi-omics analysis. GraphPad Prism 9.2 (GraphPad Software Inc., San Diego, CA, USA) was used for data visualization and figure formatting. For all analyses, we set *p* < 0.05 as the significance level: * *p* < 0.05; ** *p* < 0.01; *** *p* < 0.001; **** *p* < 0.0001.

## 3. Results

### 3.1. Cecal Transcriptome Analysis

Via the transcriptome sequencing of cecal tissue samples, we detected a total of 1,236,294,350 clean reads across all 26 samples, with an average of 47,549,782.69 clean reads per sample. The clean reads had a GC content of 46.73% and an AT content of 53.27%. Among these, 1,125,764,607 mapping events occurred, with an average mapping rate of 91.59% (RNA-Seq raw data summary was presented in Table S2). The raw sequence data reported in this paper were deposited in the Genome Sequence Archive [19] in National Genomics Data Center [20], China National Center for Bioinformation/Beijing Institute of Genomics, Chinese Academy of Sciences (GSA: CRA019017). These are publicly accessible at https://ngdc.cncb.ac.cn/gsa.

**Figure 1:**
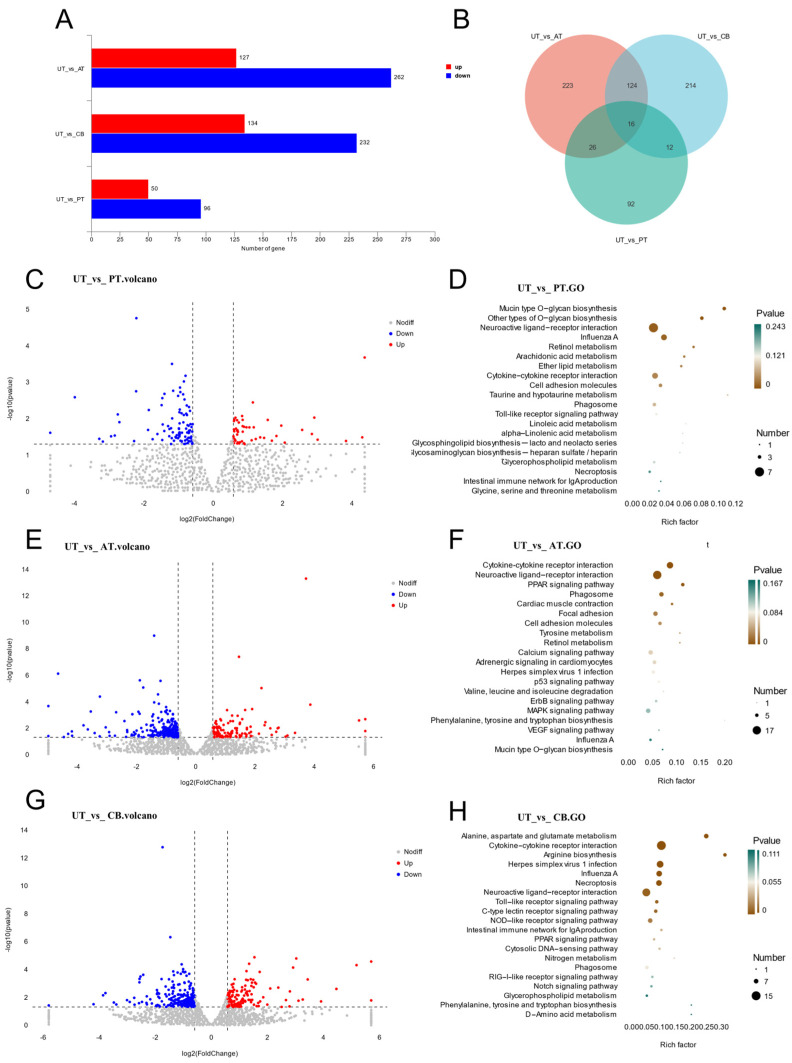
Transcriptome analysis results. (**A**): Number of differentially expressed genes; (**B**): Venn diagram of differentially expressed genes; (**C**): volcano plot of UT vs. PT; (**D**): KEGG enrichment pathways of UT vs. PT; (**E**): volcano plot of UT vs. AT; (**F**): KEGG enrichment pathways of UT vs. AT; (**G**): volcano plot of UT vs. CB, (**H**): KEGG enrichment pathways of UT vs. CB.

To investigate the impact of additives in the supplemented groups on gene expression, we performed differential expression analysis between each supplemented group and the control group. The results showed that, compared to the UT, 146 genes were differentially expressed in the PT, including 50 upregulated and 96 downregulated genes; 389 genes were differentially expressed in the AT, including 127 upregulated and 262 downregulated genes; and 366 genes were differentially expressed in the CB, including 134 upregulated and 232 downregulated genes (Figure 1A). Compared to the UT, the three treatment groups shared 16 common DEGs. The PT had 92 unique DEGs, the AT had 223 unique DEGs, and the CB had 214 unique DEGs (Figure 1B). DEGs are listed in Table S3. The volcano plots illustrating the DEGs for the three treatment groups are shown in Figure 1C,E,G.

In the PT, we identified the differential expression of IGF2BP1 (*p* < 0.05) and BMP2 (*p* = 0.017), which may be associated with growth performance in broilers. The upregulated expression of genes such as TLR4 (*p* < 0.05), IL12A (*p* < 0.05), and TREM2 (*p* < 0.05) suggests the immune-stimulating effect of *Polygonatum sibiricum*. Additionally, the differential expression of oxidative stress-related genes, including NCF1C (*p* < 0.05), indicates that *Polygonatum sibiricum* may contribute to antioxidant stress responses in broilers. In the AT, we observed the differential expression of GH (*p* < 0.01) and IGFBP2 (*p* < 0.05), which may be linked to growth performance in broilers. The differential expression of immune-related genes such as IFNG (*p* < 0.05), TNFRSF18 (*p* < 0.01), and IL17REL (*p* < 0.05) suggests that *Acer truncatum* exhibits immunomodulatory effects. Furthermore, the differential expression of oxidative stress-related genes, including HSPB1 (*p* < 0.05) and NRG1 (*p* < 0.01), implies that *Acer truncatum* may play a role in alleviating oxidative stress in broilers. In the CB, we identified the differential expression of GH (*p* < 0.01) and PDK4 (*p* < 0.01), which are associated with growth and energy metabolism in broilers. The differential expression of immune-related genes such as CXCL13 (*p* < 0.01) and IFNG (*p* < 0.05) highlights the immunomodulatory potential of *Clostridium butyricum*. Moreover, the differential expression of oxidative stress-related genes, including NOX1 (*p* < 0.05), suggests that *Clostridium butyricum* may contribute to antioxidant stress responses in broilers.

**Figure 2:**
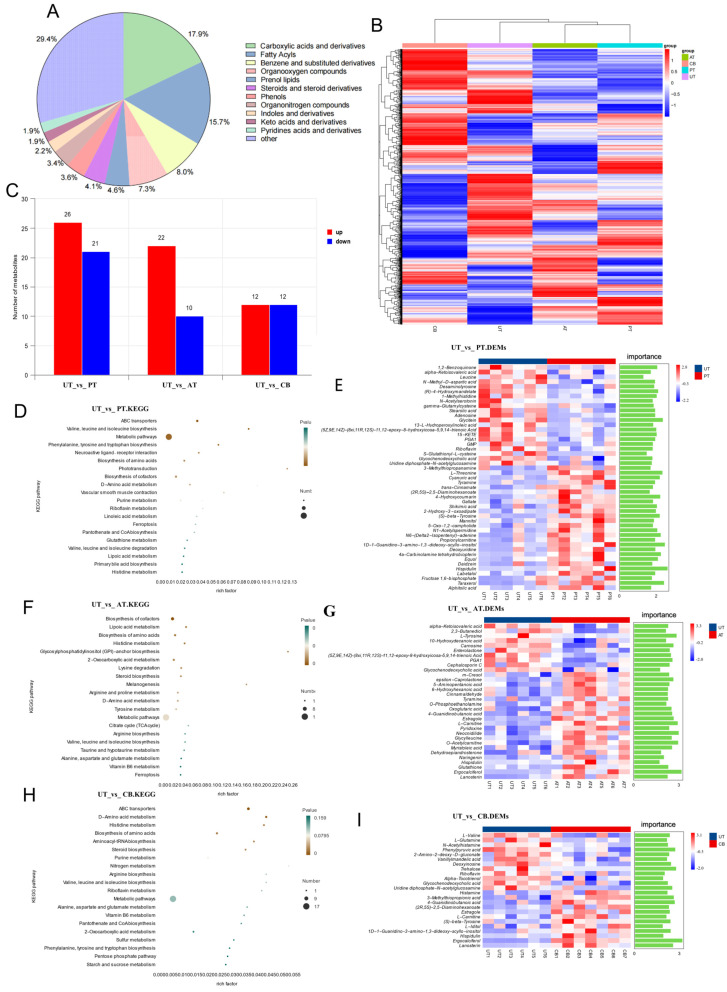
Metabolomic analysis results. (**A**): Metabolite classification; (**B**): heatmap of inter-group substances; (**C**): differential metabolite count; (**D**): UT vs. PT KEGG pathways; (**E**): UT vs. PT differential substances and VIP; (**F**): UT vs. AT KEGG pathways; (**G**): UT vs. AT differential substances and VIP; (**H**): UT vs. CB KEGG pathways; (**I**): UT vs. CB differential substances and VIP.

Compared to UT, KEGG pathway analysis revealed the existence of enriched pathways in the treatment groups (Table S4). Compared to the UT, we observed the significant enrichment of the cytokine–cytokine receptor interaction pathway and neuroactive ligand–receptor interaction pathway in all three treatment groups (Figure 1D,F,H). The retinol metabolism pathway was significantly enriched in PTs and ATs (Figure 2D,F). The influenza A pathway was significantly enriched in PTs and CBs (Figure 1D,H), and the PPAR signaling pathway was significantly enriched in ATs and CBs (Figure 1F,H). In the PT, the unique arachidonic acid metabolism pathway and cell adhesion molecules pathway are associated with immune function, while the neuroactive ligand–receptor interaction pathway is related to the growth performance of broilers. Additionally, the arachidonic acid metabolism pathway is also linked to the antioxidant stress response in broilers (Figure 1D). Similarly, in the AT, the unique cardiac muscle contraction pathway may influence the growth performance of broilers, the tyrosine metabolism pathway may be related to the antioxidant stress response in broilers, and the phagosome pathway is closely associated with the immune response (Figure 1F). In the CB, the unique pathways, such as the Toll-like receptor signaling pathway, the C-type lectin receptor signaling pathway, and the NOD-like receptor signaling pathway, and the intestinal immune network behind IgA production are closely related to immune recognition and response. Additionally, the pathways for alanine, aspartate and glutamate metabolism, arginine biosynthesis, and nitrogen metabolism are closely associated with the growth performance of broiler chickens (Figure 1H).

These results suggest that PT, AT, and CB treatments significantly influence immune function, growth performance, and oxidative stress-related pathways in JIC.

### 3.2. Cecal Metabolomic Analysis

Metabolomic analysis explored changes in the cecal metabolic profiles after supplementation with different additives. The three highest chemical classifications represented across groups were carboxylic acids and derivatives (17.9%), fatty acyls (15.7%), and benzene and substituted derivatives (8.0%) (Figure 2A). The data reported in this paper were deposited in the OMIX, China National Center for Bioinformation/Beijing Institute of Genomics, Chinese Academy of Sciences (https://ngdc.cncb.ac.cn/omix: accession no. OMIX007411 accessed on 12 October 2021) [19,20].

We constructed a metabolite clustering heatmap which strikingly demonstrated that the CB exhibited the most distinct metabolic profile and was clearly segregated from the other groups (Figure 2B). Compared to the control group, 47 differential metabolites were identified in the PT, with 26 upregulated and 21 downregulated; 32 differential metabolites were identified in the AT, with 22 upregulated and 10 downregulated; and 24 differential metabolites were identified in the CB, with 12 upregulated and 12 downregulated (Figure 2C). All DEMs are presented in Table S5.

We performed a KEGG pathway analysis on the differentially expressed metabolites; all differential metabolic pathways are presented in Table S6. The DEM identified between the UTs and PTs is closely associated with specific metabolic pathways. Leucine and valine are linked to the valine, leucine, and isoleucine biosynthesis pathways. Tyramine and trans-Cinnamate are related to the phenylalanine, tyrosine, and tryptophan biosynthesis pathways. N-Methyl-D-aspartic acid is associated with the neuroactive ligand–receptor interaction pathway. Gamma-Glutamylcysteine is connected to the ABC transporter pathway (Figure 2D,E).

In the comparison between the UTs and ATs, we observed that differently expressed m-Cresol, alpha-Ketoisovaleric acid, and Oxoglutaric acid are linked to the biosynthesis of amino acids. Tyramine and L-tyrosine are connected to tyrosine metabolism. 5-Aminopentanoic acid and O-Phosphoethanolamine are associated with arginine and proline metabolism. L-Carnitine and O-Acetylcarnitine are involved in lipoic acid metabolism. Estragole and dehydroepiandrosterone are related to steroid biosynthesis. Neocnidilide and Hispidulin are linked to melanogenesis. Glutathione is associated with glycosylphosphatidylinositol (GPI) anchor biosynthesis. Additionally, metabolites like 10-Hydroxydecanoic acid and Lanosterin are connected with the biosynthesis of cofactors and general metabolic pathways (Figure 2F,G).

In the comparison between the UTs and CB, we found that the DEMs are associated with several key metabolic pathways. Histamine and N-Acetylhistamine are linked to histidine metabolism. L-valine and L-glutamine are involved in the biosynthesis of amino acids. 4-Guanidinobutanoic acid and (S)-beta-tyrosine are associated with D-amino acid metabolism. Estragole and Lanosterin are connected to steroid biosynthesis. L-Carnitine is involved in ABC transporters, while Uridine diphosphate-N-acetylglucosamine is associated with aminoacyl-tRNA biosynthesis. We also identified metabolites such as Riboflavin and Ergocalciferol (Figure 2H,I).

Also, compared to the UT, the ABC transporter pathway was significantly enriched in both the PTs and CBs (PT: *p* < 0.01; CB: *p* < 0.01). The metabolic pathways were significantly enriched in the PTs and ATs (PT: *p* < 0.05; AT: *p* < 0.05). Several overlapping enriched pathways were identified in both the ATs and CBs, including the biosynthesis of amino acids (AT: *p* < 0.01; CB: *p* < 0.05), histidine metabolism (AT: *p* < 0.05; CB: *p* < 0.05), steroid biosynthesis (AT: *p* = 0.0228; CB: *p* < 0.05), and D-amino acid metabolism (AT: *p* < 0.05; CB: *p* < 0.01).

### 3.3. Cecal Microbial Community Structure and Function Analysis

**Figure 3:**
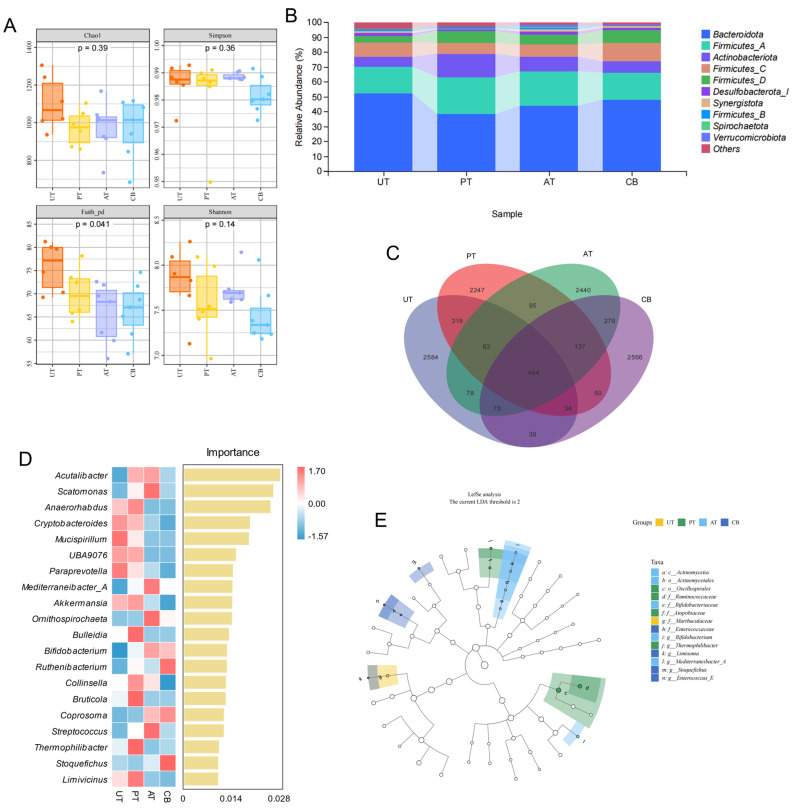
16S analysis results. (**A**): Four α-diversity indices; (**B**): phylum-level microbial composition; (**C**): Venn diagram of inter-group ASV counts; (**D**): genus-level random forest analysis; (**E**): LEFSe analysis results.

To investigate the effects of *Pulsatilla chinensis*, *Acer truncatum*, and *Clostridium butyricum* on the gut health of JIC, we analyzed the cecal 16S microbiota in each group. In assessing alpha diversity across samples, we found the Chao1, Shannon, and Faith PD indices were lower in all treatment groups compared to the UT. Among these, the difference in the Faith PD index was statistically significant (*p* < 0.05). The Simpson index was lowest in the CB, but the differences were not significant (Figure 3A). Overall, there were no significant differences in alpha diversity measures among the groups. The reduced alpha diversity indices suggest the existence of a more specialized and less complex microbial community in the treatment groups. This reduction in microbial diversity could indicate selective pressure from the treatments, potentially promoting the growth of beneficial bacteria while suppressing potentially harmful microorganisms.

Beta diversity analysis revealed that, based on Bray–Curtis distance and PCoA, there was no clear separation between the UTs and PTs, nor between the ATs and CBs. However, both the ATs and CBs showed distinct separation from the UT (Figure S2), suggesting that the addition of *Acer truncatum* and *Clostridium butyricum* may have altered the gut microbiota composition, potentially influencing gut health.

Differences in gut microbiota among groups were investigated. At the phylum level, Bacteroidota, Firmicutes, and Actinobacteriota dominated, with Bacteroidota decreasing and Firmicutes and Actinobacteriota increasing in treatment groups (Figure 3B). We used Venn diagrams to visualize the core and unique species among the groups (Figure 3C). The random forest analysis of feature importance at the genus level revealed that, in the UT, the abundances of Mucispirillum and Paraprevotella were relatively higher. In the PT, the abundances of Anaerorhabdus, Bulleidia, Bruticola, and Thermophilibacter were increased. In the AT, the abundances of Scatomonas, Mediterraneibacter_A, Ornithospirochaeta, and Streptococcus were significantly elevated. In the CB, the abundances of Ruthenibacterium, Coprosoma, and Stoquefichus were markedly increased. The increased Mucispirillum in UT may be associated with mild inflammation, while the increased Ruthenibacterium in CB and increased Mediterraneibacter_A in AT could be beneficial, but this requires further investigation (Figure 3D). The numbers of microbial taxa at the phylum and genus levels are listed in Supplementary Table S7.

Additionally, LEfSe was performed to identify taxa driving the differences between groups. At the class level, Actinomycetia was identified as a key class contributing to the intersample differences. At the order level, Oscillospirales was found to be a significant order driving the intersample differences. At the family level, Ruminococcaceae, Bifidobacteriaceae, Atopobiaceae, Muribaculaceae, and Enterococcaceae were revealed as important families contributing to the intersample differences. At the genus level, Actinomycetia was identified as a key genus driving the intersample differences. Notably, Muribaculaceae was found to be the primary family responsible for the differences between the UT and the other groups (Figure 3E).

### 3.4. Association Between 16S and Metabolome

**Figure 4:**
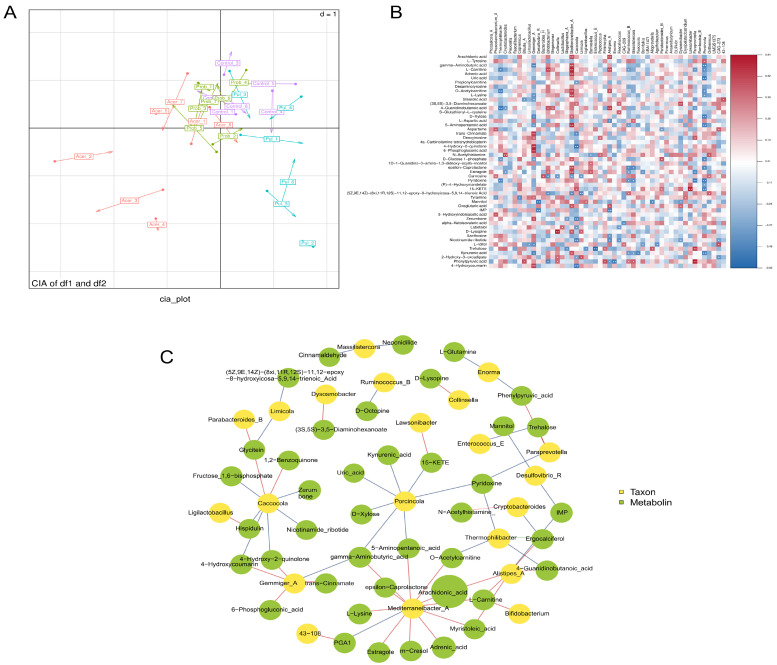
Correlation analysis between 16S rRNA sequencing and metabolomics data. The two-dimensional Co-Inertia Analysis (CIA) ordination plot. (**A**): Displays 16S rRNA data points as circles and metabolomic data as arrows in heatmap. (**B**): Illustrates the correlations between bacterial taxa and metabolites, while the association network diagram. (**C**): Represents relationships between nodes, with red edges indicating positive correlations and blue edges denoting negative correlations. * *p* < 0.05, ** *p* < 0.01.

CIA revealed a moderate overall correlation between the metabolomics and microbiomics datasets (RV coefficient = 0.4395, *p* = 0.06), indicating a certain level of association between the two. In the CIA plot, a distinct separation was observed between the AT and PT along the first axis. The UT is primarily clustered in the first quadrant, while the PT is mainly concentrated in the second quadrant. The AT is predominantly distributed across the second and third quadrants, and the CB is primarily scattered in the first and fourth quadrants. Samples from the ATs and PTs tend to suggest a separation trend (Figure 4A). We conducted Spearman correlation analysis between microorganisms and metabolites. Labetalol showed a positive correlation with Lactobacillus, while Bifidobacterium exhibited positive correlations with L-Carnitine, 4-Guanidinobutanoic acid, and Estragole, but negative correlations with Carnosine, (5Z,9E,14Z)-(8xi,11R,12S)-11,12-epoxy-8-hydroxyicosa-5,9,14-trienoic acid, and Phenylpyruvic acid. Additionally, Bacteroides_H demonstrated positive correlations with (5Z,9E,14Z)-(8xi,11R,12S)-11,12-epoxy-8-hydroxyicosa-5,9,14-trienoic acid and Phenylpyruvic acid, but negative correlations with Ruminococcus_B, L-Carnitine, 5-Aminopentanoic acid, epsilon-Caprolactone, Estragole, Pyridoxine, and Kynurenic acid. These findings suggest the existence of a close link between gut microbiota composition and metabolite profiles, influencing chicken growth and immune function (Figure 4B).

Correlation network analysis revealed the association ofCaccocola, Mediterraneibacter_A, Alistipes_A, and Gemmiger_A with a diverse range of metabolites, implicating their involvement in multiple physiological processes within the gut, including metabolic pathways, inflammatory responses, and lipid metabolism. These associations suggest the potential influence of these microbial species on gut health. Notably, elevated levels of arachidonic acid were observed, indicating its potential as a key metabolic factor contributing to gut health dysregulation (Figure 4C).

### 3.5. Association Between Transcriptome and Metabolome

**Figure 5:**
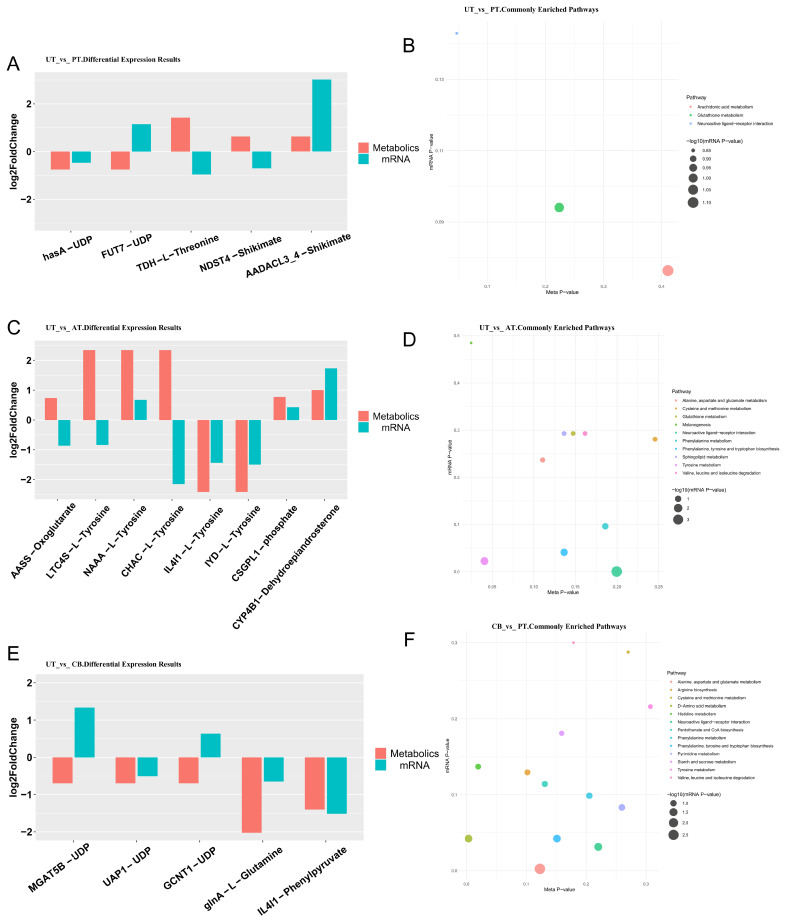
Integrative analysis of transcriptomics and metabolomics data. (**A**): Differential expression of metabolites and associated transcripts in UT vs. PT comparison. (**B**): Commonly enriched pathways in UT vs. PT differential analysis. (**C**): Differential expression of metabolites and associated transcripts in UT vs. AT comparison. (**D**): Commonly enriched pathways in UT vs. AT differential analysis. (**E**): Differential expression of metabolites and associated transcripts in UT vs. CB comparison. (**F**): Commonly enriched pathways in UT vs. CB differential analysis.

In the PT, hasA-UDP, FUT7-UDP, TDH-L-Threonine, NDST4-shikimate, and AADACL3_4-shikimate are differentially expressed (Figure 5A). The commonly enriched KEGG pathways in transcriptional metabolism are arachidonic acid metabolism, glutathione metabolism, and neuroactive ligand–receptor interactions (Figure 5B).

In the AT, AASS-oxoglutarate, LTC4S-L-tyrosine, NAAA-L-tyrosine, CHAC-L-tyrosine, IL4I1-L-tyrosine, IYD-L-tyrosine, CSGPL1-phosphate, and CYP4B1-dehydroepiandrosterone are differentially expressed (Figure 5C). The commonly enriched KEGG pathways in transcriptional metabolism include alanine, aspartate, and glutamate metabolism; cysteine and methionine metabolism; glutathione metabolism; melanogenesis; neuroactive ligand–receptor interaction; phenylalanine metabolism; phenylalanine, tyrosine, and tryptophan biosynthesis; sphingolipid metabolism; tyrosine metabolism; and valine, leucine, and isoleucine degradation (Figure 5D).

In the CB, MGAT5B-UDP, UAP1-UDP, GCNT1-UDP, glnA-L-glutamine, and IL4I1-phenylpyruvate are differentially expressed (Figure 5E). The commonly enriched KEGG pathways in transcriptional metabolism include alanine, aspartate, and glutamate metabolism; arginine biosynthesis; D-amino acid metabolism; cysteine and methionine metabolism; histidine metabolism; neuroactive ligand–receptor interaction; pantothenate and CoA biosynthesis; phenylalanine metabolism; pyrimidine metabolism; phenylalanine, tyrosine, and tryptophan biosynthesis; starch and sucrose metabolism; tyrosine metabolism; and valine, leucine, and isoleucine degradation (Figure 5F).

### 3.6. Growth Performance Outcomes

All three additives improved key growth parameters compared to the untreated group, with Clostridium butyricum showing the most pronounced effects on live body weight and muscle development (Figure S1). Detailed biometric measurements are provided in Supplementary Table S1.

## 4. Discussion

The use of Antibiotic Growth Promoters in animal feeds has been prohibited in the European Union and China, leading to a surge in research on alternative substances. Herbal medicines [21], probiotics [22], peptides [23], and organic acids [24] have become well-known as natural feed additives in broiler farming.

TCM and their extracts have been reported to show positive effects on broiler chicken production. TCM contains various bioactive components, such as polysaccharides [25], flavonoids [26], sterols, glycosides, alkaloids, terpenoids, phenylpropanoids, and polyketides [27]. These components offer multiple benefits, promoting growth by increasing body weight and feed efficiency, enhancing immune function through improved antibody production and cytokine regulation, and improving gut health by supporting microbial balance and strengthening intestinal barrier function [25].

Probiotics are beneficial active microorganisms that could improve intestinal microecological balance [28], enhance animal immunity [29], and increase the feed utilization efficiency [30]. Commonly used probiotics in broiler production include lactic acid bacteria, Bacillus, and Bifidobacterium. Probiotics can improve broiler growth performance and health status through various mechanisms, such as producing antimicrobial substances, competitively excluding harmful bacteria, and regulating immune responses [31]. Lactic acid bacteria can improve broiler growth performance, intestinal barrier health, gut microbiota balance, and immune protection [32,33].

The doses of additives were selected based on recommended commercial levels and supporting evidence from the literature. The 1% dose of Pulsatilla powder aligns with the recommended level for commercial products, which has been validated in various poultry farming practices to significantly improve growth performance and gut health, exhibiting high safety. For *Acer truncatum*, the 3% dose was chosen based on previous studies demonstrating its efficacy in enhancing performance and antioxidant capacity, with benefits such as an increased egg production rate (7.04%, *p* < 0.05) and improved plasma antioxidant activity (*p* < 0.05) in Taihang hens [34]. Similarly, the 1% dose of *Clostridium butyricum* was selected based on the recommended level for commercial probiotic products. Given the extended growth cycle of the native chickens used in this study, we relied on these established doses without conducting preliminary experiments, as they have been widely validated in poultry research and practice.

The composition of gut microbiota reflects intestinal health. Reports have shown that *Acer truncatum*, when used as a feed additive, can modulate the gut microbiota of broilers. It has enhanced antioxidant capabilities and exhibits anti-inflammatory effects [10]. *Clostridium butyricum* has been found to improve the productivity and feed efficiency of broilers [12], but there is no existing literature on the effects of *Pulsatilla chinensis* on broiler productivity. This study comprehensively discusses and compares the effects of three additives on the productivity and gut microbiota of local chicken breeds, and elucidates the underlying molecular mechanisms of these effects through transcriptomic and metabolomic analyses, aiming to provide a rational assessment of the addition of herbal medicines and probiotics.

We observed that, at the phylum level, the relative abundance of the Bacteroidota phylum decreased in all treatment groups, while that of the Firmicutes and Actinobacteriota phyla increased. The increase in Firmicutes may be associated with enhanced energy-harvesting capabilities. Some studies suggest that bacteria of the Firmicutes phylum are more adept at breaking down complex carbohydrates, potentially leading to increased energy extraction from food by the host [35]; the rise in Actinobacteriota may be linked to probiotic effects, as this phylum includes many genera considered beneficial, such as Bifidobacterium [36]. Therefore, the addition of additives may increase the abundance of beneficial bacteria in the gut, improving energy metabolism in broilers. In the Pulsatilla treatment group, the abundances of Anaerorhabdus, Bulleidia, and Thermophilibacter genera increased. Thermophilibacter is a thermophilic bacterium, and its increase may indicate a change in intestinal temperature, thereby altering the energy metabolism process in broilers. In the *Acer truncatum* treatment group, the genera Scatomonas, Mediterraneibacter_A, Ornithospirochaeta, and Streptococcus increased significantly. Mediterraneibacter_A is typically associated with cellulose degradation and the production of short-chain fatty acids [37], suggesting that the addition of *Acer truncatum* may increase cellulose intake in broilers, thereby enhancing the concentration of short-chain fatty acids in the digestive tract and improving energy utilization efficiency. The improved growth performance in ATs and CBs aligns with evidence that gut microbiota regulate muscle development via metabolic pathways [38]. The prolonged supplementation of *Clostridium butyricum* and *Acer truncatum* likely enhanced the colonization of Firmicutes and Actinobacteriota, counteracting dysbiosis-induced muscle suppression [39]. Enriched taxa may restore the key pathways essential for muscle growth, as shown in microbiota-depleted models [38].

Alpha diversity showed that the Chao1, Shannon, and Faith’s PD indices in the treatment groups were all lower than those in the UT, indicating that the treatments may have led to a reduction in microbial diversity. The difference in Faith’s PD index was statistically significant (*p* < 0.05), suggesting that the treatments not only influenced the richness and distribution of species but also impacted the phylogenetic diversity of the microbial community. This implies that the treatments may alter the overall community structure by affecting specific evolutionary lineages. The Simpson index was lowest in the CB, although the difference was not statistically significant. This could indicate that certain species in the CB might dominate the community, albeit not significantly. Previous research has demonstrated that *Clostridium butyricum* significantly increased the Sobs and Shannon indices (*p* < 0.05) while decreasing the Simpson index (*p* < 0.05) in broiler chickens [12]. These findings are not entirely consistent with our results, potentially due to differences in experimental conditions or subjects. However, our results remain plausible: we hypothesize that *Clostridium butyricum* may suppress the growth of specific microbes through the antimicrobial effects of its metabolites, thereby reducing overall diversity. Beta diversity analysis showed that, based on Bray–Curtis distance and PCoA analysis, there was no clear separation between the UT and PTs, nor between the AT and CBs. However, both ATs and CBs showed distinct separation from the UT, indicating that the addition of *Acer truncatum* and *Clostridium butyricum* may have caused changes in the gut microbiota, potentially impacting gut health.

Compared to the control group, the differential metabolites in the addition groups primarily involved key biological processes such as amino acid metabolism, lipid metabolism, energy metabolism, and antioxidant defense. These findings suggest that the addition of TCM and *Clostridium butyricum* modulated the gut metabolic environment, which may have implications for chicken growth and immune function. Further analysis using KEGG pathways revealed that the differential metabolites in the *Pulsatilla chinensis* addition group were involved in pathways related to amino acid metabolism and energy metabolism, consistent with studies demonstrating that Pulsatilla regulates energy metabolism and glycerolipid metabolism to improve intestinal function [40]; the *Acer truncatum* treatment involved carbohydrate metabolism and lipid metabolism, aligning with existing research findings showing that Acer truncatum reduces lipid accumulation and enhances hepatic and intestinal health through lipid oxidation [41]. The metabolites in the *Clostridium butyricum* treatment were associated with amino acid metabolism, nucleotide metabolism, and antioxidant responses. Previous studies have shown that supplementation with 1 × 10^9^ colony-forming units (cfu)/kg of *Clostridium butyricum* significantly increased the serum total antioxidant capacity (t-AOC), superoxide dismutase (SOD), and glutathione peroxidase (GSH-Px) activities in the CB at day 42 [12]. A study involving the addition of 0.3% ATL extract to the basic diet for 42 days found that ATL can increase the abundance of Bacteroidota, reduce the proportion of Firmicutes, and exert antioxidant and anti-inflammatory effects simultaneously [10]. These results are consistent with our results. In the 16S and metabolome correlation analysis, we found that Caccocola, Mediterraneibacter_A, Alistipes_A, and Gemmiger_A were correlated with multiple metabolites, involving metabolic pathways, inflammatory responses, and lipid metabolism within the gut, suggesting that these microbial species may impact intestinal health. Notably, higher levels of arachidonic acid were observed, which may be a key metabolic factor in regulating intestinal health. This is consistent with its critical role in modulating intestinal health and inflammatory responses [42].

Our study demonstrated that all three treatments shared some common DEGs, yet each treatment also exhibited unique gene expression profiles. The addition of *Pulsatilla chinensis*-enriched pathways was related to immune function (cytokine–-cytokine receptor interactions, Toll-like receptor signaling) and growth performance (glycine, serine, and threonine metabolism). The enrichment of the cytokine–cytokine receptor interaction pathway plays a crucial role in mediating inflammatory responses and immune regulation. This pathway has been shown to be associated with immune responses in diseases, such as respiratory syncytial virus infection [43] and Eimeria infection in chicken jejunum [44]. This indicates that *Pulsatilla chinensis* may enhance the immunity and growth development of broilers by modulating immune cell activity and nutrient utilization, corroborating the traditional use of herbal medicines for immune regulation and growth promotion in poultry. The addition of *Acer truncatum*-enriched pathways was related to immune function (cytokine–cytokine receptor interactions, Toll-like receptor signaling, phagosome), growth performance (glycine, serine, and threonine metabolism, taurine and hypotaurine metabolism, retinol metabolism), and oxidative stress (arachidonic acid metabolism, linoleic acid metabolism). This suggests that *Acer truncatum* may enhance immune responses, growth performance, and antioxidant capacity, consistent with previous studies [10]. The addition of *Clostridium butyricum*-enriched pathways was related to immune function (RIG-I like receptor signaling, Notch signaling), growth performance (arginine metabolism, phenylalanine, tyrosine, and tryptophan metabolism), and oxidative stress (glycerophospholipid metabolism, D-amino acid metabolism). This indicates that *Clostridium butyricum* may modulate the immune responses, growth, and redox balance of broilers, as previously documented in the literature [14,45].

In the Pulsatilla group, we observed an increase in L-aspartate levels and alterations in quercetin metabolism. L-aspartate plays a crucial role in the urea cycle and purine metabolism [46], while quercetin possesses antioxidant and anti-inflammatory properties [47]. The accumulation of these metabolites suggests that nitrogen metabolism and antioxidant capacity may have improved. Additionally, changes in the neuroactive ligand–receptor interaction pathway indicate enhanced neuroendocrine regulation, which may be crucial for improving poultry’s health. The neuroactive ligand–receptor interaction pathway is critical for neurotransmission and the regulation of neuronal activity. This pathway has been shown to be associated with apparent metabolizable energy in chickens [48] and egg production in laying hens [49]. The Acer truncatum group showed significant changes in phosphoethanolamine metabolism, which could affect membrane lipid composition—a critical component in cell signaling and adaptation to environmental stress [50]. Changes in 2-oxoglutarate and related enzymes suggest alterations in energy metabolism, beneficial for maintaining cellular homeostasis under stressful conditions [51]. Interestingly, both the ATs and CBs exhibited downregulation of the tyrosine metabolism pathway. Tyrosine is a precursor to several neurotransmitters and hormones, and its metabolism is closely linked to stress responses [52]. This change indicates a reduction in environmental stressors. In the ATs and CBs, the upregulation of dehydroepiandrosterone levels and cytochrome P450 4B1 enzyme suggests alterations in steroid hormone metabolism. Steroid hormones play a key role in stress responses and immune function in poultry [53]. In all treatment groups, we observed changes in enzymes related to glutathione metabolism. Glutathione is an antioxidant, and these changes may reflect the additives’ role in clearing oxidative stress in the body.

Despite the small sample size per group, a key limitation of our study, we conducted comprehensive multi-omics analyses on local breed chickens, including 16S rRNA sequencing, transcriptomics, and metabolomics. These high-dimensional data provided sufficient statistical power to support our conclusions. To address this limitation, we conducted a thorough analysis of the factors related to sample statistical power and sample size. By leveraging advanced statistical methods and rigorous data analysis, we were able to mitigate the potential impact of the small sample size on our results. Looking forward, larger sample sizes and additional multi-omics studies on local breed chickens will further validate our findings and provide deeper insights into the underlying mechanisms. Additionally, our study did not evaluate the potential side effects of herbal and probiotic additives. Although some studies suggest that the prolonged use of herbal additives may lead to liver or kidney damage [54,55], the short rearing cycle of broilers likely reduces the risk of such effects. Furthermore, there is currently no evidence that these additives negatively affect broiler product quality. Nevertheless, future studies should include biochemical and histopathological assessments to further investigate the safety of these additives. Another potential limitation of this study is the use of the Jianmen-guan breed, a slow-growing native breed from Sichuan Province known for its unique slaughter value as a local poultry breed, which may have unique physiological or genetic characteristics that could influence the results. Future studies should include multiple breeds to evaluate the generalizability of the findings.

While the current study focused on the individual effects of *Pulsatilla chinensis*, *Acer truncatum*, and *Clostridium butyricum*, the potential synergistic interactions among these additives remain unexplored. Previous studies have demonstrated that combining phytobiotics and probiotics can enhance their efficacy compared to individual use [56]. For example, the anti-inflammatory and antimicrobial properties of *Pulsatilla chinensis* might synergize with the gut microbiota-modulating effects of *Clostridium butyricum*, potentially leading to improved growth performance and gut health. However, further research is needed to validate these potential synergies and optimize their combined application in poultry production. Our future research may systematically evaluate the synergistic effects of combined *Pulsatilla chinensis* and *Clostridium butyricum* administration in poultry, with a focus on identifying their optimal compatibility ratios to enhance gut health and production performance.

The inclusion of *Pulsatilla chinensis*, *Acer truncatum*, and *Clostridium butyricum* in the diet demonstrated the potential to improve the growth performance of JIC. These feed additives also contributed to reshaping the cecal microbiota composition, which is crucial for maintaining gut health. Furthermore, they promoted the secretion of beneficial metabolites, which are known to play a critical role in supporting intestinal integrity and overall productivity. In terms of improving production performance, the addition of *Acer truncatum* and *Clostridium butyricum* significantly enhanced the growth performance of chickens. Pulsatilla showed the most pronounced effect in increasing the proportion of beneficial gut bacteria and reducing the abundance of harmful bacteria.

The costs and availability of the tested additives are important considerations for their practical implementation. *Pulsatilla chinensis* is a traditional herbal medicine with wide availability, while *Acer truncatum* is a locally sourced species that may face challenges in long-distance transportation. *Clostridium butyricum*, as a commercial probiotic product, is cost-effective and readily accessible, making it a feasible option for broader application. The application of herbal additives and probiotics in commercial poultry production should primarily consider factors such as cost-effectiveness, stability under storage conditions, and adaptability to different farming systems. Our study provides preliminary evidence supporting the potential of *Pulsatilla chinensis*, *Acer truncatum*, and *Clostridium butyricum* as viable alternatives to antibiotics, but further research is needed to validate their efficacy and feasibility in large-scale commercial settings. Overall, *Pulsatilla chinensis* showed the most promising potential as an antibiotic alternative due to its remarkable effects on gut health and immune function improvement in chickens, warranting further investigation for its application in poultry production.

## 5. Conclusions

This study provides evidence that the strategic use of *Acer truncatum*, *Clostridium butyricum*, and *Pulsatilla chinensis* as feed additives can positively reshape the gut microbiota, enhance metabolic efficiency, and bolster the immune and antioxidative systems of chickens. These natural alternatives hold promise for sustainable poultry production by improving productivity and health without relying on antibiotics, thereby contributing to a more resilient and eco-friendly agricultural practice. Further studies are warranted to validate these findings under diverse farming conditions and to elucidate additional benefits and potential synergies when combining these additives.

## Figures and Tables

**Table 1:** Composition and nutritional content of the basal diet.

Ingredient	Content (%)	Nutritional Indicator
Corn	55.0	Dry Matter (DM)
Soybean Meal	30.0	Crude Protein (CP)
Fish Meal	5.0	Crude Fat (EE)
Wheat Bran	5.0	Crude Ash
Limestone	1.5	Neutral Detergent Fiber (NDF)
Dicalcium Phosphate	1.0	Acid Detergent Fiber (ADF)
NaCl	0.3	Metabolizable Energy (ME)
Fat	1.2	
Compound Premix	1.0	

## Data Availability

The data reported in this paper have been deposited in the OMIX, China National Center for Bioinformation/Beijing Institute of Genomics, Chinese Academy of Sciences (https://ngdc.cncb.ac.cn/omix: accession no. OMIX007411 accessed on 18 September 2024) and China National Center for Bioinformation/Beijing Institute of Genomics, Chinese Academy of Sciences (GSA: CRA019017).

## Supplementary Materials

The following supporting information can be downloaded at: https://www.mdpi.com/article/10.3390/ani15091262/s1, Table S1: Slaughter Performance Measurement. Table S2: Summary of RNA-Seq Raw Data. Table S3: Differential Gene Expression Analysis Results. Table S4: Enriched KEGG of DEGs between three groups. Table S5: DEMs between three groups. Table S6: Enriched KEGG of DEMs between three groups. Table S7: Statistical table of the number of microbial taxa at each level. Figure S1: PCoA of Beta Diversity Based on 16S rRNA Gene in Cecal Content. Figure S2: β-diversity of cecal microbiota.
