# Supplementary material for: Preliminary Multi-Omics Insights into Green Alternatives to Antibiotics: Effects of Pulsatilla chinensis, Acer truncatum, and Clostridium butyricum on Gut Health and Metabolic Regulation in Chickens
Source: Animals (Basel). 2025 Apr 29;15(9):1262. doi: 10.3390/ani15091262 (PMC12071075; doi:10.3390/ani15091262)
Supplement: Supplementary file 1 [file animals-15-01262-s001.zip › Fig S1.pdf]

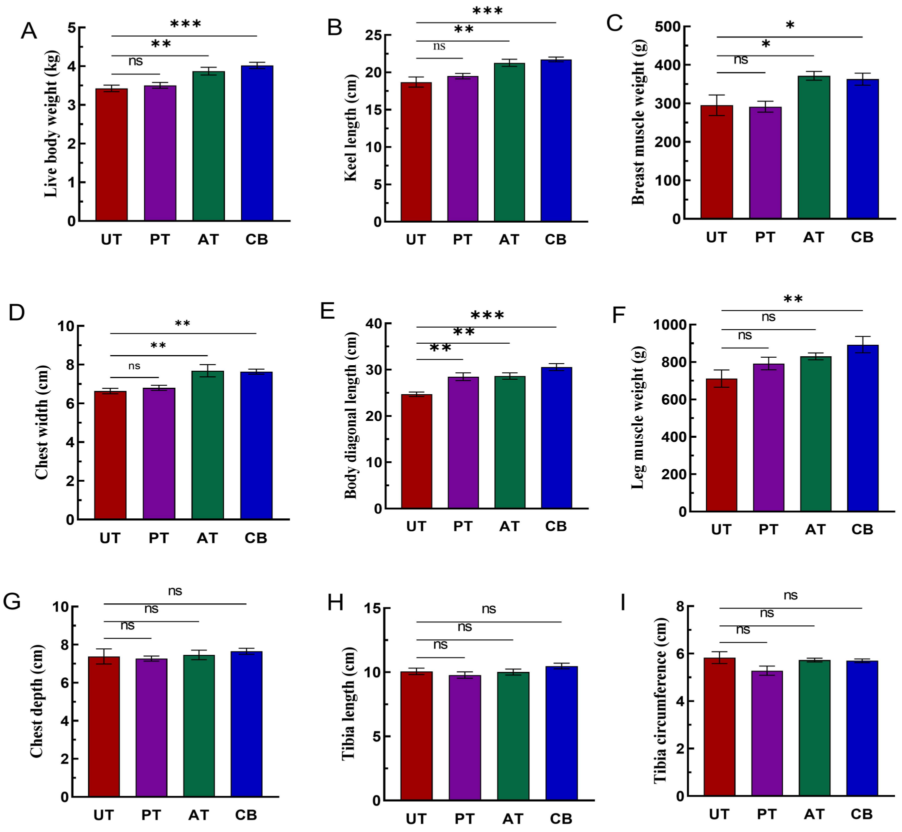

Figure S1. Slaughter trait measurements. **(A)**: live body weight, **(B)**: keel length, **(C)**: breast muscle weight, **(D)**: chest width, **(E)**: body diagonal length, **(F)**: leg muscle weight, **(G)**: chest depth, **(H)**: tibia length, **(I)**: tibia circumference.
