# Supplementary figures and images for: Preliminary Multi-Omics Insights into Green Alternatives to Antibiotics: Effects of Pulsatilla chinensis, Acer truncatum, and Clostridium butyricum on Gut Health and Metabolic Regulation in Chickens
Source: Animals (Basel). 2025 Apr 29;15(9):1262. doi: 10.3390/ani15091262 (PMC12071075; doi:10.3390/ani15091262)

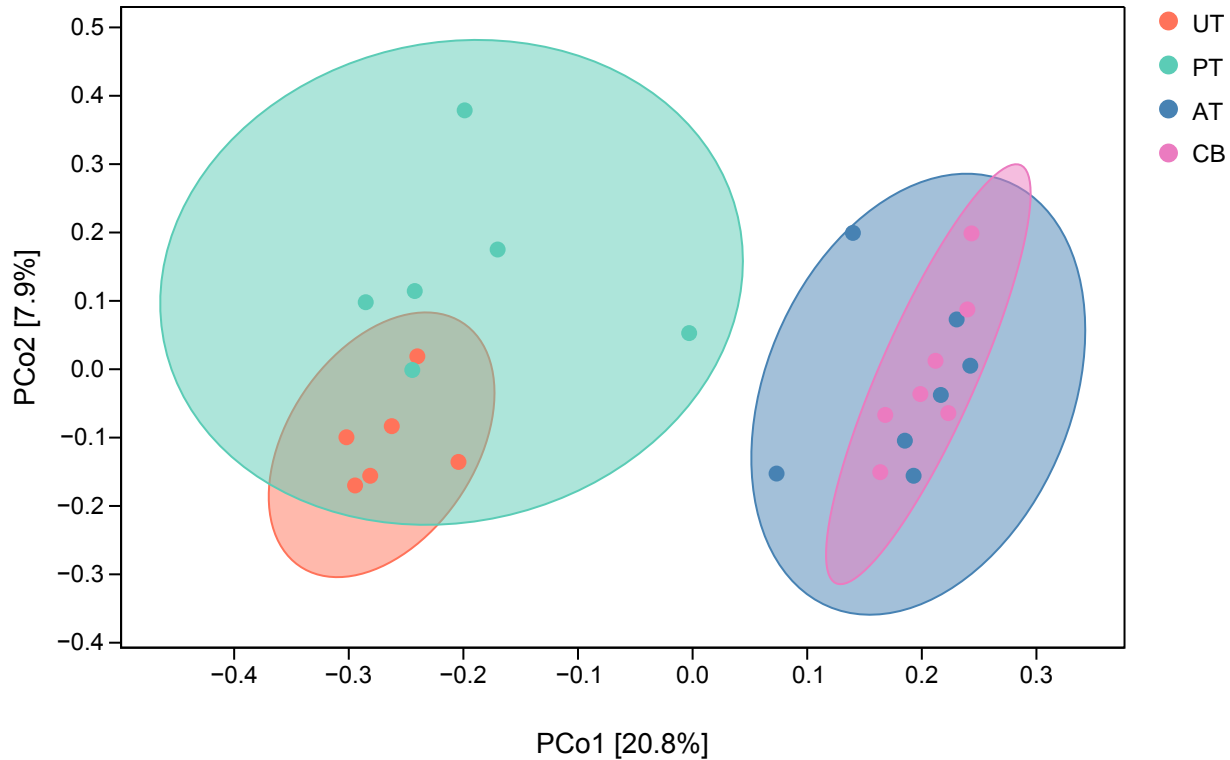

Supplement: Supplementary file 1 [file animals-15-01262-s001.zip › Fig S2.pdf]
